# A brain-inspired sequence learning model based on a logic

**DOI:** 10.1038/s41598-025-97777-8

**Published:** 2025-04-19

**Authors:** Bowen Xu

**Affiliations:** Department of Computer and Information, Temple University, Philadelphia, PA 19122 USA

**Keywords:** Sequence learning, Non-axiomatic logic, Brain-inspired, Mini-column, Computational science, Computer science

## Abstract

Sequence learning is a crucial aspect of intelligence research, with sequence prediction tasks commonly used to evaluate the performance of sequence learning models. This paper introduces and tests a novel sequence learning model that mimics the structure of neocortical mini-columns and is grounded in *Non-Axiomatic Logic*, offering interpretability. The model’s learning mechanism encompasses three steps: hypothesizing, revising, and recycling, enabling it to operate effectively under conditions of *insufficient knowledge and resources*. The model’s performance is assessed using synthetic datasets for sequence prediction. The results demonstrate that the model consistently achieves high accuracy across various levels of difficulty, reaching the theoretical maximum. Furthermore, the model’s concept-centered representation effectively avoids catastrophic forgetting, a finding supported by the experimental results.

## Introduction

*Sequence leaning* (sometimes known as *sequential learning*, *serial order learning*, etc.) refers to acquiring the proper ordering of events or stimuli^1,2^. It is the foundation of many learning processes for an intelligent agent to interact with the world, such as sensorimotor perception, natural language acquisition, etc. It is widely investigated in Cognitive Science and Artificial Intelligence (AI). This paper aims to propose a biologically-plausible, logic-based model for sequence learning.

In Cognitive Science, the Serial Reaction-Time task is commonly used to measure subjects’ performance in sequence learning^3^. In this task, subjects’ reaction times decrease over time when exposed to repeated sequences of stimuli. In contrast, in AI, the performance of sequence learning models is often evaluated based on their anticipation accuracy. AI tasks for assessing sequence learning models include sequence *prediction*, *generation*, *recognition*, and *decision making*^2^. Various approaches for sequence learning have been proposed, such as Markovian methods^4^, recurrent neural networks^5^, etc. Neural networks, including the Transformer model^6^, have made significant advances in natural language processing, which can be seen as a specific instance of sequence learning tasks. Among biologically plausible models, Hierarchical Temporal Memory (HTM) is particularly intriguing: it models neocortical columns to memorize frequently occurring sequences; however, handling uncertainty remains a challenge for the HTM model^7^.

*Explainability* is a crucial issue in AI safety, and a major criticism of neural networks is their lack of transparency: these models often operate as black or grey boxes, making it difficult for developers to understand their internal workings and address unexpected behaviors. One approach to addressing this issue is to ensure that a model adheres to a logical framework. In other words, a model is considered interpretable if it can be described through logical representation. Beyond well-known frameworks such as First-Order Predicate Logic (FOPL) and Expert Systems, *Non-Axiomatic Logic* (NAL) is a promising model of intelligent reasoning^8^. NAL effectively handles uncertainty and has proposed solutions to the symbol grounding problem^9,10^, a long-standing challenge in AI research that questions whether machines truly understand the meaning behind symbols. NAL includes logical rules for temporal inference^11^, such as *deduction* and *induction*: Future events are predicted based on current knowledge and *deduction*, while new knowledge is acquired through *induction*. These rules offer a scaffold for an AI system to logically understand event sequences. However, extracting temporal patterns from sequences using this logical representation remains a challenging problem.

Previous research indicates that the mini-column is a widely distributed structure in the neocortex^12^. The brain’s structure may offer insights into how machines can learn patterns from sequences; for instance, a mini-column in the neocortex can respond to a specific event (e.g., event *X*), while an individual neuron within the mini-column responds to that event in a particular sequence (e.g., event *X* in context *WXYZ*)^7^.

Building on these ideas, the proposed model incorporates the structure of mini-columns while simultaneously utilizing logical representation. In this model, Non-Axiomatic Logic ensures *interpretability* by representing a connection between two neurons as a *temporal* *implication* or *equivalence* with an associated *truth value*. The strength of a connection is adjusted through *temporal induction*, while future neuronal activations are predicted using *temporal deduction*. Thanks to the properties of NAL^8,13^, the model is inherently capable of managing uncertainty, and its behaviors and internal workings are fully comprehensible to human observers.

Since the model can be described as a spiking neural network and interpreted through logic, it is further termed a *conceptual network*. This network is a graph where concepts are represented as nodes, and the relations between these concepts are represented as links. Concepts are dynamically constructed as new types of events and are introduced into the network. The conceptual network begins empty, with new links being created and revised in real time based on the inference rules of NAL and the learning mechanisms proposed in this paper. The total memory available is constrained, which limits the number of links to a theoretical maximum. To manage this constraint, redundant links are recycled during the runtime.

The model is tested on *prediction* tasks, where the input consists of a list of events, and the model is expected to predict future events. The list is assumed to be infinite, with no defined beginning or end (although, in practice, starting and ending point are usually necessary), meaning the model cannot theoretically memorize all the contents. With this assumption, the learning procedure must be “*online*” and “*life-long*”^14^. For example, consider the input events “$(...,\$, A,B,C,D, \$,\$,X,B,C,Y,\$,...)$”, where “$\$$” represents a random event and the characters denote different types of events. The types of events are not predetermined before system initialization but are dynamically constructed by the model. In this example, there are two prototypes of sequences: “(*A*, *B*, *C*, *D*)” and “(*X*, *B*, *C*, *Y*)”. This means that the sequence “(*A*, *B*, *C*)” is always followed by event *D*, whereas, after observing (*B*, *C*)” without other information, either *D* or *Y* might occur subsequently. The “Results” section explores various lengths and numbers of prototypes to test the model’s capacity. Additionally, *catastrophic forgetting*^15^, which refers to the phenomenon where a neural network quickly loses previously learned knowledge when trained on new data, is a significant challenge in models with distributed representations (e.g., neural networks). Qualitative results indicate that the proposed model effectively avoids catastrophic forgetting.

This research underscores the potential benefits of applying logical approaches to sequence learning by demonstrating how models based on Non-Axiomatic Logic can address key challenges in sequence prediction. The incorporation of *concept-centered representation*, combined with the model’s ability to handle uncertainty and avoid catastrophic forgetting, showcases the advantages of using logic over traditional *distributed representation*. The model’s strong interpretability, stemming from its basis in NAL, enables clearer understanding and debugging of the model’s internal processes, making it a potentially valuable tool for practical applications. Furthermore, the model’s capabilities of adapting to continuously evolving sequences and learning in real time highlight its potential for real-world scenarios where patterns in data streams are changing with time. These attributes not only validate the effectiveness of logical approaches but also pave the way for future research into more sophisticated and interpretable models for complex sequence learning tasks.

**Fig. 1:**
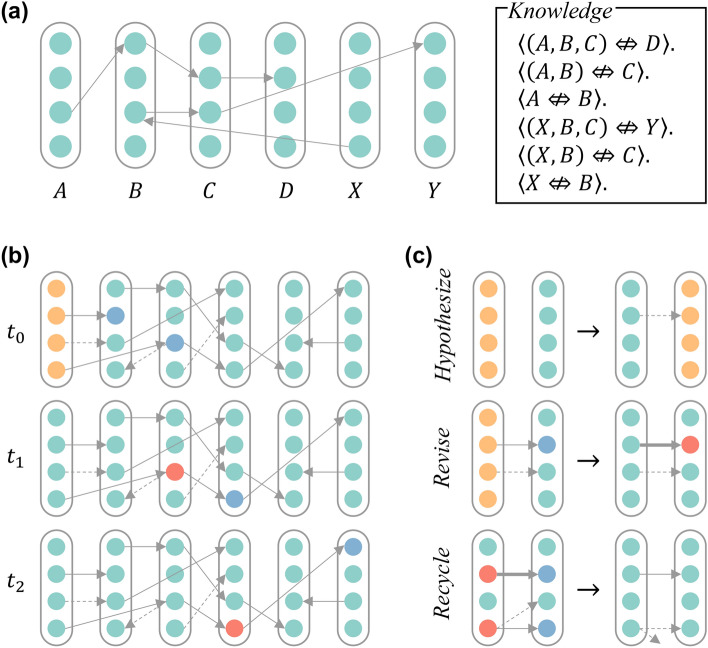
Illustrations of the model—**(a)** An example of the learned network. There are six *concepts*, *A* through *D*, *X*, and *Y*. Each *concept* is represented as a *column* that contains multiple *nodes*. There are *links* between *nodes*. Multiple *links* constitute a *chain*, representing a group of knowledge. For instance, *chain* “$(A^{(3)}, B^{(1)}, C^{(2)}, D^{(2)})$” corresponds to three beliefs (represented by logical expressions), “$\langle (A, B, C) \mathrel {\Leftrightarrow \!\!\!\!\!\!/ \ }D \rangle .$”, “$\langle (A, B) \mathrel {\Leftrightarrow \!\!\!\!\!\!/ \ }C \rangle .$”, and “$\langle A \mathrel {\Leftrightarrow \!\!\!\!\!\!/ \ }B \rangle .$”. **(b)** An example of inference procedure. It shows the internals of the model at three consecutive time-steps. At $t_0$, *concept* *X* is activated. Since there is no context, all the *nodes* are activated. It anticipates $A^{(2)}$ and $B^{(3)}$ to occur for the next time-step. *Node* $A^{(2)}$ is not anticipated even if there is a *link* from $X^{(3)}$ to $A^{(3)}$, because the *truth-value* (i.e., the strength of the *link*) is too low. At $t_1$, *concept* *B* is activated. *Node* $B^{(3)}$ is activated due to the anticipation, while the other *nodes* in *B* remain silent. In the meantime, $C^{(3)}$ is anticipated. Similarly, at $t_2$, $C^{(3)}$ is activated, and $Y^{(1)}$ is anticipated. **(c)** The learning mechanism. When there is no *link* between two *concepts*, some *links* are built as *hypotheses*. When one or two *nodes* at both ends of a *link* are activated, the *truth-value* in the *link* is revised according to distinct situations. When the number of *links* exceeds a threshold, one or some of them are deleted.

## Approach overview

To design a model that is both explainable and comprehensible to human beings, a promising way is to ground it in logic. Specifically, Non-Axiomatic Logic (NAL)^8^ emerges as a noteworthy candidate. NAL operates under the assumption of *insufficient knowledge and resources* (AIKR), enabling a system to dynamically acquire and revise knowledge. To learn the temporal order of events in sequences, a fundamental inference rule involved in NAL is *temporal induction*: when events $\textit{press-button}$ and $\textit{get-food}$ occurs successively, it can be inferred that “*press-button* implies *get-food*” (formally “$\textit{press-button} \mathrel {\Rightarrow \!\!\!\!\!\!\!/ \ }\textit{get-food}$”), with the occurrence time of $\textit{press-button}$ preceding that of $\textit{get-food}$. This process is supported by the inference rule

1 $$\begin{aligned} \{ A ~\langle f_1;c_1\rangle , C ~\langle f_2; c_2\rangle \} \vdash A \mathrel {\Rightarrow \!\!\!\!\!\!\!/ \ }C ~\langle F_{ind}(\langle f_1;c_1\rangle , \langle f_2,c_2\rangle ) \rangle , \end{aligned}$$

where *A* and *C* can be replaced by any concrete terms, while $\langle f; c\rangle$ denotes the *truth-value* of a statement. The function $F_{ind}(\cdot )$ in NAL maps two *truth-values* into a composite one. The first component, *f*, in *truth-value* is called *frequency*, which reflects the ratio of positive evidence ($f=w^+/w$, where $w^+$ quantifies positive evidence, and *w* denotes total evidence comprising both positive evidence $w^+$ and negative evidence $w^-$, with $w=w^++w^-$). The the second component, *c*, is called *confidence*, which is a measure of total evidence ($c=w/(w+k)$, where *k* is a constant and typically 1.0). Both *f* and *c* are real numbers between 0 and 1, and they jointly reflect the uncertainty in knowledge. Applying $F_{ind}(\cdot )$, the total evidence in the conclusion’s *truth-value* does not exceeds 1.0: specifically, $w^+ = f_1f_2c_1c_2$, and $w=f_2c_1c_2$, which are subsequently converted to *f* and *c*. In contrast, a definite belief typically involves tens or hundreds of pieces of evidence. Consequently, conclusions derived through the *induction* rule are inherently *weak* and best interpreted as *hypotheses*.

A system may observe the pattern that event *C* consistently follows event *A*. For each observation, the induction rule generates a statement of the form “$A \mathrel {\Rightarrow \!\!\!\!\!\!\!/ \ }C ~\langle f_i; c_i\rangle$”. Multiple statements sharing the structure “$A \mathrel {\Rightarrow \!\!\!\!\!\!\!/ \ }C$” but differing in their *truth-values* $\langle f_i; c_i\rangle$ can be consolidated into an overall belief through the *revision* rule in NAL,

2 $$\begin{aligned} \{ S ~\langle f_1;c_1\rangle , S ~\langle f_2; c_2\rangle \} \vdash A \mathrel {\Rightarrow \!\!\!\!\!\!\!/ \ }C ~\langle F_{rev}(\langle f_1;c_1\rangle , \langle f_2,c_2\rangle ) \rangle , \end{aligned}$$

where *S* can be substituted with any concrete term (e.g., “$A \mathrel {\Rightarrow \!\!\!\!\!\!\!/ \ }C$”), while *truth-value* function $F_{rev}(\cdot )$ aggregates the positive and negative evidence of the two premises: the positive evidence of the conclusion, $w^+$, is calculated by $w^+=w^+_1+w^+_2$ , whereas the the negative evidence, $w^-$, is given by $w^-=w^-_1+w^-_2$, and $\langle f; c \rangle$ can be converted from $w^+$ and $w^-$. As more evidence accumulates, a *hypothesis* would becomes increasingly definite, making it more reliable for predicting future events through *deduction*.

Given prediction $A \mathrel {\Rightarrow \!\!\!\!\!\!\!/ \ }C$ and the occurrence of event *A*, the *deduction* rule in NAL can be applied to forecast the occurrence of event *C*,

3 $$\begin{aligned} \{ A \mathrel {\Rightarrow \!\!\!\!\!\!\!/ \ }C ~\langle f_1;c_1\rangle , A ~\langle f_2; c_2\rangle \} \vdash C ~\langle F_{ded}(\langle f_1;c_1\rangle , \langle f_2,c_2\rangle ) \rangle , \end{aligned}$$

where $F_{ded}(\cdot )$ calculates the *truth-value* as follows: $f=f_1f_2$, and $c=f_1f_2c_1c_2$. If there is no evidence for *A*’s occurrence, *or* if the predictive relationship between *A* and *C* is too weak, the *confidence* in the anticipation of *C*’s occurrence remains very low, implying that the system is uncertain for *C*’s occurrence. Only if both of the premises are true to a large degree will the anticipation be reliable.

Statement “$A \mathrel {\Rightarrow \!\!\!\!\!\!\!/ \ }C ~\langle f; c\rangle$” represents the fundamental form of temporal knowledge in a system’s memory. From the perspective of discrete mathematics, a *concept*—marked by a term, e.g., “*bank*”, “*press-button*”, “*A*”, to name a few—corresponds to a node (or vertex), while the relationships between concepts correspond to links (or edges). In this manner, the system’s memory is organized as a *graph*, or more precisely, a *conceptual network*.

While the theoretical background of NAL^8^ described above provides essential context for the proposed model, representing temporal relations involving multiple events in sequence learning remains considerably more complex; organizing inference steps that enable a system to learn sequential patterns is, at present, an unresolved challenge. For instance, a sequence like “$\textit{green-light}, \textit{press-button}, \textit{get-food}$” cannot be represented as a simple linear chain of vertices, as an event may have multiple causes and effects. Effective prediction of an event’s outcome necessitates consideration of prior contextual events. For example, given the knowledge “$\textit{green-light} \mathrel {\Rightarrow \!\!\!\!\!\!\!/ \ }\textit{press-button} \mathrel {\Rightarrow \!\!\!\!\!\!\!/ \ }\textit{get-food}$” and “$\textit{red-light} \mathrel {\Rightarrow \!\!\!\!\!\!\!/ \ }\textit{press-button} \mathrel {\Rightarrow \!\!\!\!\!\!\!/ \ }\textit{get-hurt}$”, it is impossible to determine whether event “$\textit{get-food}$” or “$\textit{get-hurt}$” will occur given merely the occurrence of “$\textit{press-button}$”. However, if event “$\textit{gree-light}$” previously occurred, the system can confidently predict the occurrence of “$\textit{get-food}$”. This illustrates that future event anticipation is highly context-dependent.

Drawing inspiration from the mini-column structure of the neocortex^16^ and prior work on modeling mini-columns and spiking neural networks^7,17,18^, this sequence learning model enables the system to discern temporal regularities among events. In this model, a *column* is interpreted as a *concept*, with *nodes* within a *column* being context-sensitive and functioning as aliases of the *concept*. Intuitively, the relationship between a *column* and a *node* is analogous to the relationship between a word and a notion it conveys. For example, the word “bank” may refer to different meanings in “bank of America” versus “the bank of the river”. The system similarly predicts subsequent concepts based on the context-dependent interpretation of the current concept.

Utilizing this columnar structure (see Fig. 1a), the system can effectively distinguish between varying contexts. For instance, suppose the system has learned two pieces of knowledge “$A^{(3)} \mathrel {\Rightarrow \!\!\!\!\!\!\!/ \ }B^{(1)} \mathrel {\Rightarrow \!\!\!\!\!\!\!/ \ }C^{(2)} \mathrel {\Rightarrow \!\!\!\!\!\!\!/ \ }D^{(2)}$” and “$X^{(4)} \mathrel {\Rightarrow \!\!\!\!\!\!\!/ \ }B^{(3)} \mathrel {\Rightarrow \!\!\!\!\!\!\!/ \ }C^{(3)} \mathrel {\Rightarrow \!\!\!\!\!\!\!/ \ }Y^{(1)}$”, where $T^{(i)}$ represents the *i*-th *node* in *column* *T*. Upon the occurrence of event *X*, the system anticipates $B^{(3)}$. Subsequently, when event *B* transpires, the absence of anticipation $B^{(1)}$ leads the system to infer that $B^{(2)}$ is the contextually appropriate notion. Consequently, $C^{(3)}$ and $Y^{(1)}$ will be anticipated and validated through future experience.

When an event is input, the system employs the *deduction* rule (see Eq. 3, or Eq. 9) to predict subsequent events. These anticipations directly influence the selection of concepts processed in the next timestep (via Eq. 12). If a concept, represented by a *node*, was anticipated, it becomes more likely to be processed upon the occurrence of its corresponding event (see Fig. 1b for illustration).

A major challenge in the model’s design lies in learning *node* chains that represent knowledge of sequential events. Due to AIKR^19^, it is unacceptable for intelligent systems to consider all possible event combinations, particularly when the length of event stream is extensive or even infinite. To address this, the model adheres to two key constraints: (1) the capacity of memory should a constant, and (2) the processing time for an input event should not exceed a small constant. Under these constraints, each item in memory undergoes three stages throughout its life cycle, birth, survival, and death. At the system level, these stages correspond to the learning procedure’s three steps, *hypothesizing*, *revising*, and *recycling* (illustrated in Fig. 1c).

During the *Hypothesizing* step, when an event is input, the system constructs a *concept*—represented as a *column* containing a constant number of *nodes*—in the runtime and seeks for potential *cause*(s). A cause could also be an unknown one, meaning that there is no prediction link between the cause and the current event, but they occur close to each other in time. In this case, the system has to speculate a concept as the cause by evaluating *nodes*’ *utilities* (see Eq. 13), forming a new *link* that could be utilized later. A newly constructed *link* possesses a weak *truth-value*, thereby serving as a *hypothesis*. A cause could also be a known one, if there is a prediction link from that concept to the current one, and the antecedent *concept* was recalled previously. In this case, the system picks out a *link* with the highest value (see Eq. 15 for details) in the *Revising* step. During the *revising* step, links are strengthened or weakened according to system’s experience. Once a *link* is selected, it is updated according to the *induction* rule (see Eq. 1, or Eqs. 6–8) and the *revision* rule (see Eq. 2, or Eq. 10). Useful links—those with strong *truth-values*—stabilize over time, persisting within the memory. Conversely, during the *Recycling* step, due to that the total number of links connected to or from a concept is maintained at a constant level, useless links (assessed by Eq. 17) must be pruned to free up memory resources, ensuring the system retains only the most valuable information.

In each working cycle, the system handles an input event by selecting a few *nodes*, applying *deduction* to forecast future events, and updating some *links* through the *hypothesizing*, *revising*, and *recycling* steps described above. Each input event activates a single corresponding *column*, which contains a small, constant number of *nodes*; each *node* is associated with a small, limited number of *links*. Thus, the processing time for each working cycle remains a small constant approximately. Importantly, irrelevant knowledge outside the actively processed concept remains unaffected. This localized processing mechanism underpins the model’s capability of continual learning (see “Results” section).

Each working cycle comprises several reasoning steps, each of which is governed by an inference rule—*deduction*, *induction*, or *revision*—whose validity and rationality is justified by the NAL theory^8^. Therefore, in principle, an observer can explain the output of the system by checking the reasoning steps, each of which is understandable and interpretable by human beings owing to the logic.

Further details of the model are provided in “Model design” section.

## Results

The model is evaluated from two key perspectives. First, I demonstrate that the model is capable of learning multiple sequences in real time. Second, I show that it retains knowledge acquired long ago, even after incorporating newly acquired information. This second aspect is more non-trivial than it may initially appear: the predominant method in this field, deep neural networks^20^, is known to suffer from *catastrophic forgetting*^15^—wherein the model almost entirely loses previous knowledge after being trained on new tasks. For decades, catastrophic forgetting has long plagued machine learning researchers. Although some significant progress has been made in recent years, the problem has not been solved completely^21,22^. In contrast, the proposed model demonstrates robustness against catastrophic forgetting without injecting tricks, primarily due to its distinct knowledge representation—*concept-centered representation*^23^. The model’s memory updates are confined to a limited set of concepts, theoretically preventing interference with other regions of memory. This property is further validated through experimental results.

In the experiments, the model is tested using synthetic datasets. Unlike typical evaluations of machine learning methods, this approach involves no explicit separation between a training set and a test set. This is because the learning process is considered to be both *online* and *life-long*: each sample observed by the agent serves both as a training sample and a test sample. Additionally, current experiences are not required to resemble past ones, indicating that no stable data distribution is assumed. Nevertheless, an agent has to rely on its past experiences to adapt to the environment; the key requirement is that the model maintains the capability to continuously update its understanding throughout its life.

The data stream is manufactured in the following way^7^. Suppose there are $n_r$ types of events; each event is labeled with a *term*, such as characters like “*A*”, “*B*”, “*X*”, or strings like “$\textit{e0347}$”, and “$\textit{e1001}$”, which identifies the type of an *event*. Regardless of how a *term* appears to human developers, within the system, it merely names a *concept*, whose meaning depends on its acquired (rather than predetermined) relations with other *concepts*, as suggested in Non-Axiomatic Logic (NAL)^8^. A dataset in this study is a stream of *events*, which contains sequences such as “(..., *A*, *B*, *C*, *D*, ...)”, or “(..., *X*, *B*, *C*, *Y*, ...)”. Some *events* are determined by their predecessors; for instance, given a particular context, *event* “*B*” is always followed by “*C*” and preceded by either “*A*” or “*X*”; event “*D*” follows the sequence“(*A*, *B*, *C*)”, whereas given “(*B*, *C*)”, either “*D*” or “*Y*” is expected to occur subsequently. Other *events* are randomly generated, leading to the whole stream of *events* partially unpredictable. At each timestep, an *event* is generated and input to the system, and the system should predict the next *event* as accurately as possible.

With this form of input data, three aspects are considered for evaluating the model, *capacity*, *catastrophic forgetting*, and *capability*, though the capability aspect is analyzed only in theory.

### Capacity tests

Evaluating the capacity of the model is related to two factors, the number of sequences and the length of a sequence that is expected to be recognized. In a test, datasets are generated following the prototype “$(\$,..., \$, E_1,..., E_m, \$,..., \$)$”, where $E_1,..., E_m$ are deterministic events which that remain consistent in every sample of the prototype, while “$\$$” is the unpredictable variable that varies across samples. The dataset’s parameter *m* denotes the number of deterministic events in each sequence. The dataset comprises *p* prototypes. When an event occurs, the model predicts the subsequent event. If an event is anticipated and occurs subsequently, it is considered correctly anticipated. The proportion of correctly anticipated events within a given past period (e.g., the past 100 timesteps) defines the anticipation accuracy of the current timestep.

**Fig. 2:**
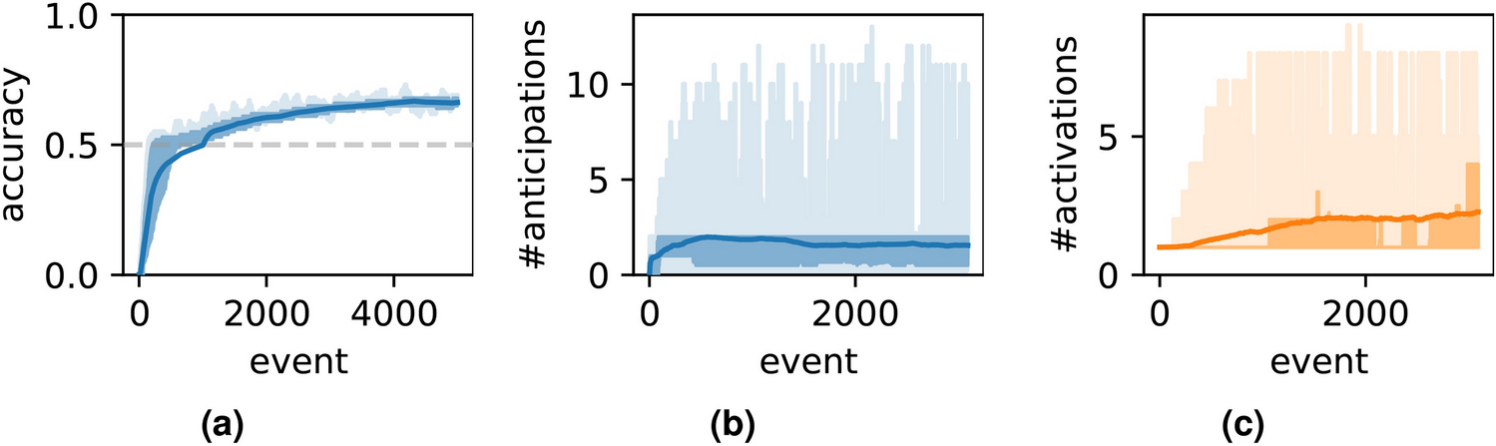
Capacity-test results for the simple case, where the prototypes of sequences are “$(\$, \$, A, B, C, D, E, \$)$” and “$(\$, \$, X, B, C, D, Y, \$)$”, where “$\$$” denotes a random event. (**a**) The accuracy of anticipation. (**b**) The number of anticipations. (**c**) The number of active nodes.

Firstly, a simple case is tested. Suppose each event is named by a single character (from *A* to *Z*), so that there are 26 possible types of events for the model. The dataset contains two prototypes of sequences “$(\$, \$, A, B, C, D, E, \$)$” and “$(\$, \$, X, B, C, D, Y, \$)$”. In this case, $m=5$ and $p=2$, with only $50\%$ of the events are deterministic and predictable. The test results are presented in Fig. 2. Figure 2a shows how anticipation accuracy evolves over time. At each timestep, multiple anticipations may occur, and Fig. 2b shows the number of events anticipated by the model. Ideally, only one event should be anticipated if the system confidently identifies the context; multiple anticipations indicate that the system considers several contexts. On average, approximately two events are anticipated at each timestep. Figure 2c shows the number of activated nodes in the model—in general, a node’s activation signifies that the model has recognized a certain context. Fewer active nodes correspond to a clearer context. As shown in Fig. 2c , approximately two nodes are activated on average per timestep.

**Fig. 3:**
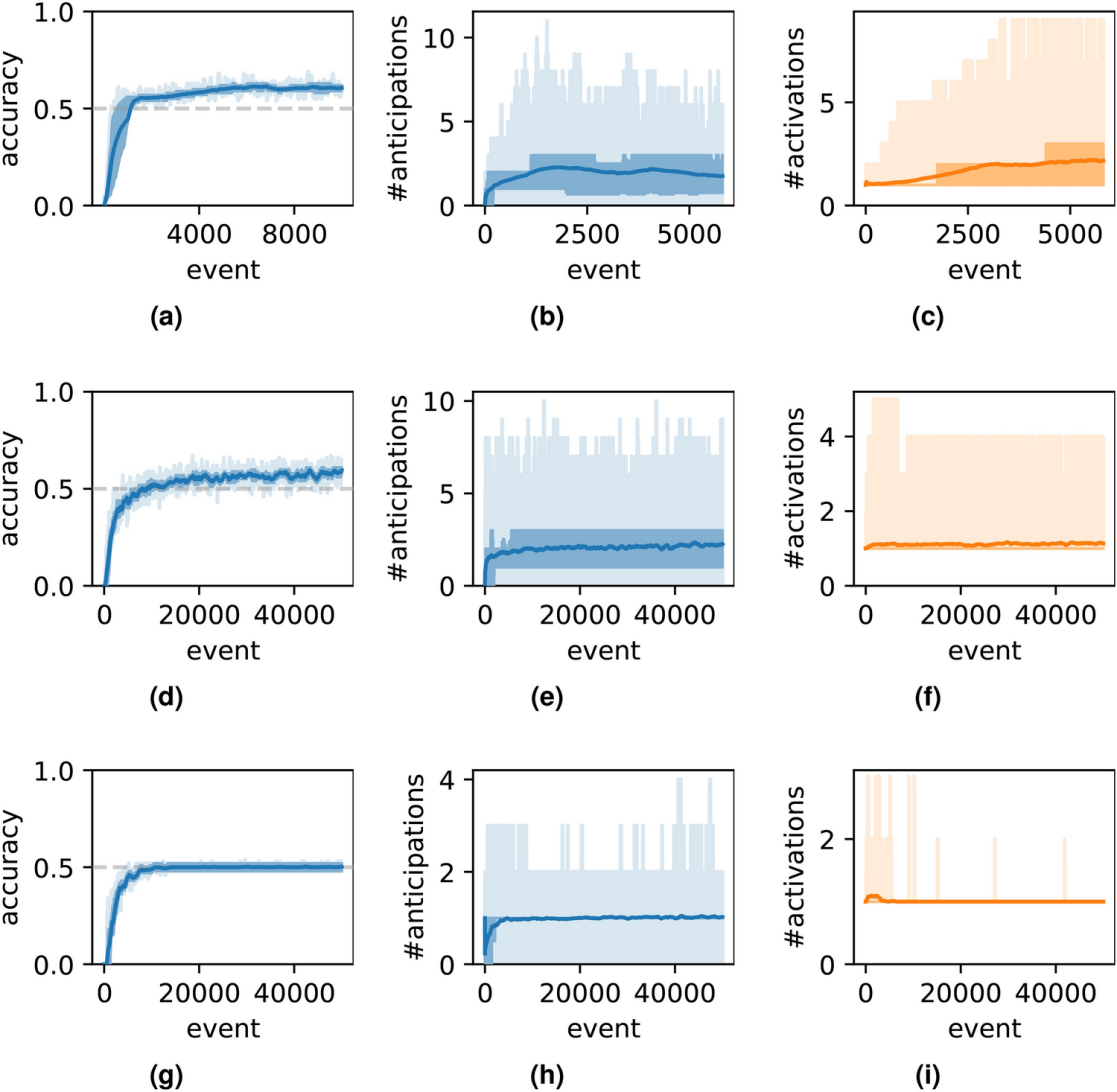
Capacity-test results with different options of length *m* and the number of prototypes *p*, and even the different numbers of types of events. (**a–c**) Are test results with $m=5$, $p=5$, and 26 types of events. (**d–f**) Are test results with $m=14$, $p=20$, and 26 types of events. (**g–i**) Are test results with $m=14$, $p=20$, and 1000 types of events.

Secondly, the model is tested under sequence lengths *m*, numbers of prototypes *p*, and different numbers of event types. In all datasets, the proportions of unpredictable events is maintained at $50\%$. As shown in Fig. 3, the model demonstrates effective anticipation of future events. With $m=5$ and $p=5$ (see Fig. 3a ), as well as $m=14$ and $p=20$ (see Fig. 3d ), the anticipation accuracy in both cases exceeds $50\%$, surpassing the theoretical maximum. Two factors may explain this phenomenon: the random component of the dataset provides a non-zero probability of correct predictions by chance, and the model partially learns patterns from the random events.

A simpler setting for the model involves increasing the number of event types beyond 26. The test results, shown in Fig. 3g–i, corresponds to a scenario where the number of types $n_r$ is 1000. The accuracy hovers around 50%, with both the number of anticipated and active nodes averaging approximately one. The model performs better in this setting than the previous ones, because in the previous tests, a single event type was likely involved in multiple sequence prototypes, potentially causing confusion. In contrast, with a significantly larger variety of event types, each type is more likely to be associated with a unique context, making it easier for the model to memorize and differentiate distinct patterns

### Catastrophic forgetting tests

The issue of *catastrophic forgetting* (also known as *catastrophic interference*) was proposed by McCloskey and Cohen in 1989^15^, highlighting that distributed representations in connectionist networks (*a.k.a.* *deep neural networks* nowadays) possess an undesirable property whereby updates with new data interfere with the memory of previously learned information, leading to substantial forgetting of previous experiences. Some modern research (e.g.^21,24^) has sought to address this issue over the years.

Since the model designed herein adopts the *concept-centered* representation—a form of local representation distinct from the distributed representation common in deep learning—this annoying property seems probably theoretically should not occur. However, due to the system’s limited memory and computational resources, as assumed in this study, it must simultaneously retain new information while potentially forgetting previously learned content. Thus, the acquisition of new knowledge may interfere with older information. It is therefore essential to assess the extent of this interference—at least qualitatively, if not quantitatively-to determine whether it reaches a catastrophic level.

**Fig. 4:**
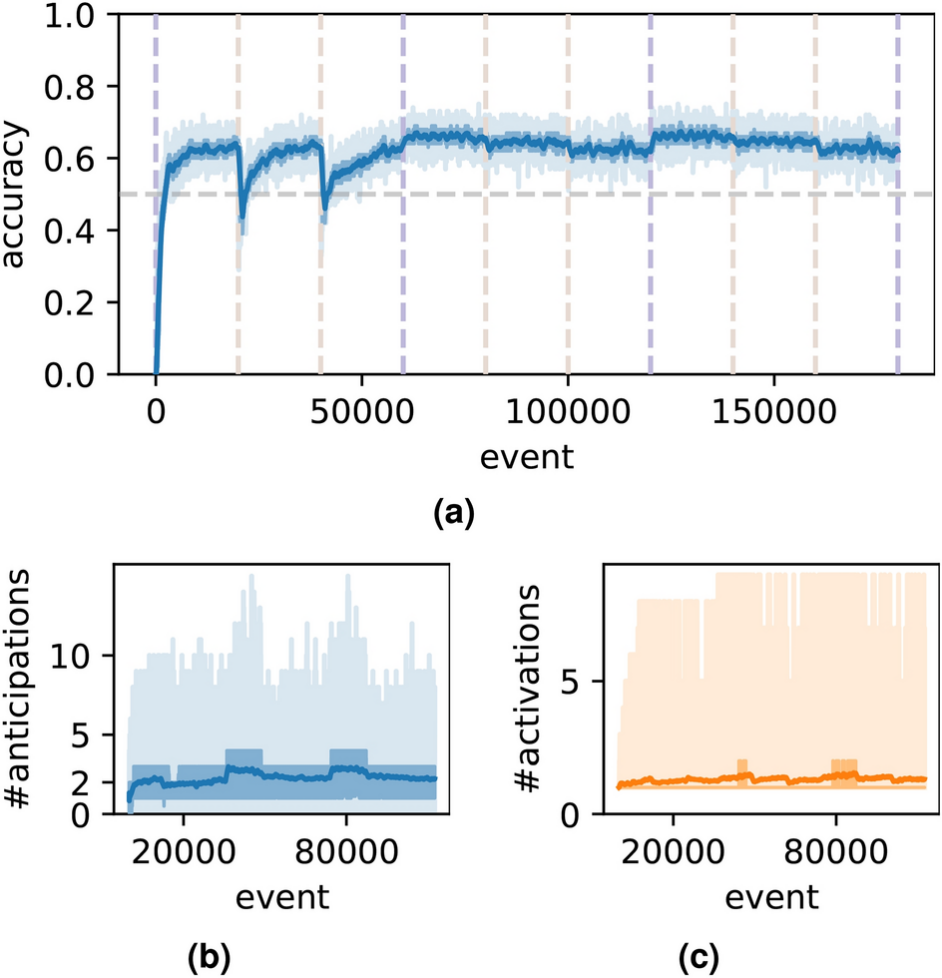
Results of catastrophic forgetting test when the number of concepts $n_r=26$.

**Fig. 5:**
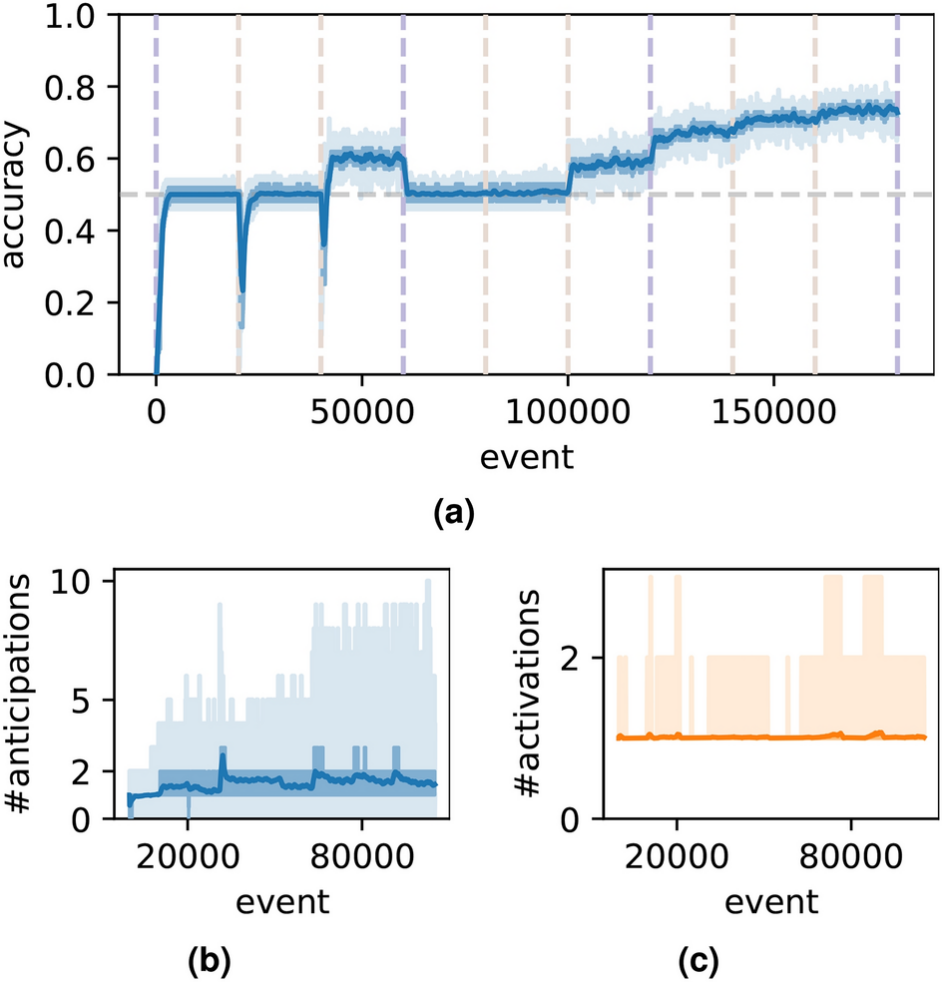
Results of catastrophic forgetting test when the number of concepts $n_r=1000$.

The test results for catastrophic forgetting are shown in Figs. 4 and 5. In each episode, 20 sequence prototypes, whose lengths are all 14, are generated, with 26 event types (in Fig. 4) or 1000 event types (in Fig. 5). The prototypes vary across different episodes. After processing three consecutive episodes, the model is re-exposed to previously encountered prototypes. If catastrophic forgetting were present, a significant decline in anticipation accuracy would be observed upon revisiting the same patterns. However, as demonstrated in Figs. 4 and 5, no such decline occurs. These findings suggest, at least qualitatively, that the model is resistant to catastrophic forgetting.

### Capability analysis

The scope of the model’s capability needs to be clarified. First, while the model lacks sufficient generality, it can be considered a preliminary step toward modeling the complex nature of intelligence. Second, the model focuses on a specific aspect of intelligence, i.e., *sequence learning*. Third, in the current design, the model handles only scenarios in which a single event occurs at each timestep; cases involving multiple simultaneous events are beyond the scope of this study. Forth, the time interval of any pair of events is assumed to be constant. This assumption enables the model to address situations where event order is important but temporal spacing is not.

Within this defined scope, the model is capable of learning patterns from an unbounded stream of events. Simultaneously, it leverages Non-Axiomatic Logic to handle uncertainty. This property (i.e., the ability to handle uncertainty) is theoretically derived, thereby requiring no experimental validation.

### Summary

In this paper, a brain-inspired model for sequence learning is proposed. The model leverages Non-Axiomatic Logic (NAL)^8^ as the foundation of representation, inference, and learning. The model is brain-inspired since it emulates the *mini-column* structure in the neocortex^12,16,25^.

Although the model is inspired by the human brain, AI systems require a clear rationale for its structural design beyond a superficial explanation such as “because the brain looks like that”. The choice to adopt the *mini-column* structure stems from its ability to represent a concept with distinct meanings across different contexts. A *neuron* within a *mini-column* is activated when part corresponding *concept*’s meaning is recalled. *Neurons* are connected as a *chain*, reflecting *concepts* organized sequentially. Due to the *Assumption of Insufficient Knowledge and Resources* (AIKR)^19^, the total number of links must not exceed a constant, necessitating a balance between memorization and forgetting. It is unacceptable to memorize the entire dataset (performing *offline learning*^14^) or all possible sequences. Meanwhile, the time complexity for processing each input event should remain constant. Based on this principle, the learning mechanism comprises three steps, *hypothesizing*, *revising*, and *recycling*. The *hypothesizing* and *recycling* steps manage resource allocation to ensure compliance with AIKR. In the *revising* step, candidate *links* are selected for temporal* induction* and *revision*, which are logical rules defined in NAL. For predicting future *events*, the temporal* deduction rule* is applied to generate anticipations. The knowledge learned by the model can be converted to *Narsese*, the formal language of NAL, enabling full explainability and trust-worthiness.

The dataset for evaluating the model is assumed to be an endless stream of events, implying that, theoretically, it lacks a defined beginning or end. Consequently, the model must perform *online learning*^14^ and operate in *real-time*. The dataset is synthetically generated, consisting of 50% predictable events and 50% random events. For the predictable portion, a specified number (denoted as *p*) of sequence prototypes are generated, each with a specified length (denoted as *m*). To validate the model’s capacity, it is tested with varying values of *m* and *p*, requiring it to anticipate subsequent events. The accuracy of these anticipations, shown in Figs. 2 and 3, demonstrates that the model performs well under different capacity demands, achieving or surpassing 50% accuracy—approximately the theoretical upper limit. Additionally, a separate task, known as *continual learning*^21^, assesses whether the model experiences *catastrophic forgetting*^15^, a persistent challenge in models with distributed representation (e.g., neural networks). As shown in Figs. 4 and  5, the proposed model does not exhibit *catastrophic forgetting*. This stems from its use of local representations (*concept-centered* representation), where modifying one *concept* or its associated connections does not disrupt unrelated *concepts*. Nevertheless, theoretically, some forgetting is unavoidable due to resource constraints.

To conclude, this paper demonstrates the potential of learning sequential patterns through a logical approach; however, further research opportunities remain that are both promising and worth exploring.

## Discussion

Several implications can be drawn from this work. First, this paper demonstrates the potential for learning sequential patterns based on a logical framework—a problem traditionally addressed by purely statistical models (e.g., Hidden Markov Models^4^), neurodynamics models (e.g., HTM^7^, spiking neural networks^18^), and neural networks (e.g., RNN^5^). The proposed model is fully interpretable through Non-Axiomatic Logic^8^, enabling human developers to explain the system’s behavior by recording and examining its internal processes in a human-understandable manner. This interpretability offers a valuable alternative to high-performing yet opaque models, particularly neural networks, which are often criticized as “black boxes”.

Second, the proposed model can be regarded as a *neural-symbolic* system^26,27^, where the basic idea is to combine symbolic methods with neural approaches. Unlike many previous works that either use neural networks to fit logical expressions or leverage symbolic knowledge to enhance neural networks, this model unifies neurons and symbols within a cohesive framework. Under this framework, a *concept* can be simultaneously interpreted as a *mini-column* or *neuron* from a biological perspective and as a logical element from a symbolic perspective. This dual interpretation provides insights into the longstanding question of how concepts emerge from neural activity in the brain, a topic extensively explored in fields such as AI, psychology, and philosophy^28–35^. These perspectives suggest that neural and logical representations may reflect different views of the same underlying entity.

Third, the learning mechanism proposed in this study can be summarized by the following principle: *Computational resources tend to converge toward knowledge with lower levels of uncertainty.* In the model, a *link* with a higher *truth-value* is more likely to be strengthened and maintained. While this principle is not novel, it serves as a valuable guideline for designing AI systems and is consistent with observations in biological systems. For instance, in neuroscience and neuronal dynamics, the well-known *winner-take-all* rule^36–38^ shares the same intuition. In psychology, Piaget’s theory suggests that new information input to a subject is incorporated into already existing knowledge^39^. In other words, the existing knowledge will be allocated computational resources to give a meaning to the content. Earlier, in Chinese traditional philosophy, as it is said in *Tao Te Ching*, “The *Way of Nature* reduces excess and replenishes deficiency. By contrast, the *Way of Humans* is to reduce the deficient and supply the excessive”. The learning process of the proposed model exactly follows the *Way of Humans*.

**Fig. 6:**
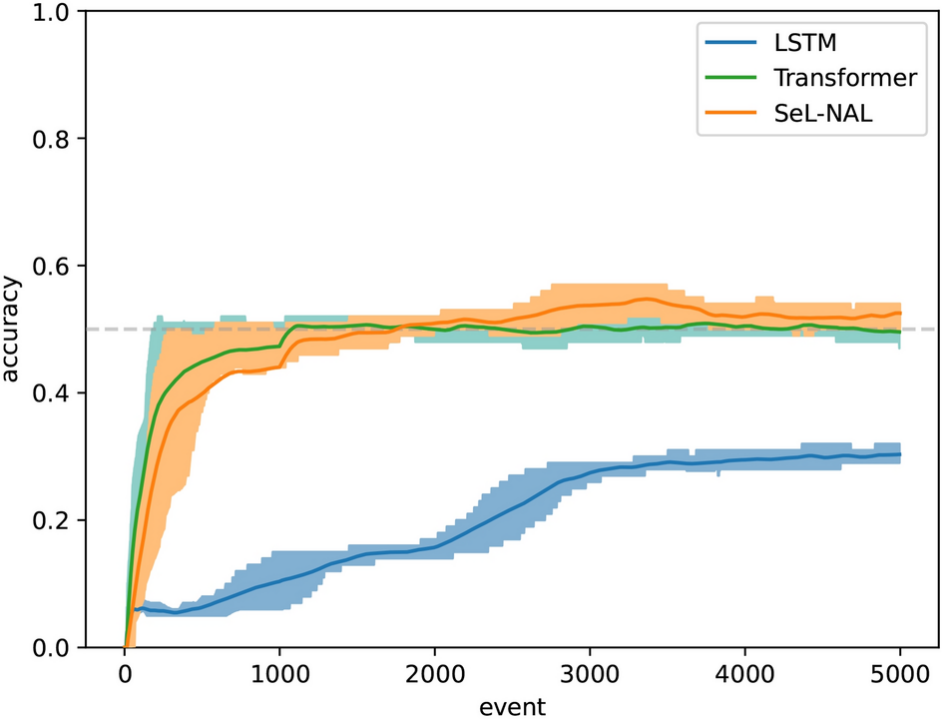
Comparison among LSTM, Transformer, and the model proposed. The prototypes to be learned are “(*A*, *B*, *C*, *D*, *E*)” and “(*X*, *B*, *C*, *D*, *Y*)”. The theoretically maximal accuracy is set at 50%.

Compared to other sequence learning models, the proposed approach is based on different theoretical assumptions. With AIKR, the types of events are unknown prior to system initialization. therefore, corresponding representations should be generated at run-time. In contrast, typical Hidden Markov Models (HMMs)^4^ require the pre-specification of event types, which cannot be modified once the system begins operation. In such models, the structure of the state transition matrix and the size of the observation set must be predetermined, preventing HMMs from handling novel events; conversely, the proposed model dynamically constructs new concepts when encountering previously unseen events. In neural networks, an event is represented by a vector, and theoretically, there is no restriction on handling new events as long as they can be represented in this form. However, the lack of explainability limits their application in certain scenarios. Under the *online* and *continual learning* settings, LSTM^40^ and Transformer^6^ are compared with this model regarding anticipation accuracy. As shown in Fig. 6, the model proposed performs as well as Transformer, while outperforms LSTM significantly. Besides, the explainability of this model is an attractive property, especially compared to neural networks. The performance of the proposed model is similar to HTM^7^, though in HTM adopts quite different theoretical foundations. There are some advantages of distributed representations in HTM, for example, robustness to noise and damage.. In contrast, the use of *concept-centered* representation enables the model to handle uncertainty naturally.

To summarize, this study introduces a novel sequence learning model, designed from the ground up under a distinct technical paradigm—one grounded in logical reasoning, specifically Non-Axiomatic Logic (NAL), and constrained by the Assumption of Insufficient Knowledge and Resources (AIKR)^19^. This approach provides a paradigm shift away from traditional statistical or neural models, offering an alternative that emphasizes interpretability, rational decision-making, and alignment with biological constraints. As such, the model should be viewed as an early exploration in a new research direction rather than a direct competitor to well-established methods.

While the model demonstrates strong interpretability and promising empirical performance—particularly in the synthesized sequence prediction tasks and its robustness against catastrophic forgetting—it is important to acknowledge certain limitations. Most notably, the current work lacks rigorous mathematical proofs to fully validate its theory. This limitation arises from the distinct theoretical assumptions underlying the model, which differ significantly from those adopted in many previous studies that often rely on idealized conditions for mathematical tractability. In contrast, the biologically plausible and logic-based framework of this model, combined with the AIKR constraints, makes the derivation of formal proofs considerably more challenging.

Despite these challenges, the model’s design is grounded in well-established logical inference rules, and experimental results consistently support its effectiveness. Nevertheless, developing formal mathematical analyses—such as convergence proofs, complexity bounds, or generalization guarantees—remains an important direction for future research. Such analyses could strengthen the theoretical underpinnings of this work and enhance its acceptance in research communities that emphasize formal guarantees.

Moving forward, future work will focus on refining the model, exploring partial theoretical guarantees under constrained settings, and expanding its applicability to more complex and real-world scenarios. Bridging the gap between theoretical rigor and practical applicability is crucial for the further development of explainable, biologically inspired, and resource-constrained sequence learning models. The potential benefits of this research path—particularly in applications demanding transparency—underscore the importance of continued exploration along this route.

## Model design

This study presents a unified model that bridges the gap between biological plausibility and conceptual interpretability. Drawing inspiration from the columnar organization of neurons and the contextual nature of concepts, the model posits an intrinsic correspondence between neural activations and logical inferences. By employing context-dependent mini-columns and adopting Non-Axiomatic Logic (NAL), a unified model is designed, facilitating human-understandable representations, and enabling effective sequence learning through mechanisms of *hypothesizing*, *revising*, and *recycling*.

### Representation

The correspondence between neurons and concepts has been a longstanding topic of discussion in neuroscience and brain science, with one of the most famous examples being the so-called “grandmother cell”—the idea that a concept is encoded by a group of neurons. This paper also aims to establish a unification of structure and principle, positing that the same model can be interpreted and represented from both neural and logical perspectives, with an intrinsic correspondence between the two. From this viewpoint, neural networks and *conceptual networks* are regarded as “two sides of the same coin,” rather than one being constructed upon the other or one emerging from the other.

#### Neural representation

Previous research in neuroscience has discovered the presence of columnar arrangements of neurons in the brain, known as *mini-columns*^16^. In a brain-inspired model, the activation of these neurons is context-dependent^7^. The proposed model adopts a similar principle. Additionally, studies on spiking neural networks has shown that neurons can exist in multiple states, including *resting* (or *non-active*), *depolarized* (or *predictive*), and *activated* (or *active*) states—Note that an elaborate framework of spiking neurons involves a more complex range of states; however, this study employs a simplified spiking neuron framework^37^ containing the essential components necessary for sequence learning. In the proposed model, a neuron reflects to the occurrence of an event, with the *depolarized* state indicating the anticipation of the event and the *activated* state indicating the event has been observed.

When an event occurs, the corresponding *mini-column* processes it by considering all possible sequences to which the event may belong. This raises question: which sequence does the current event correspond to? In other words, which neuron within the *mini-column* should be activated, given that a neuron represents a component of a specific sequence? Without contextual information, all neurons would be activated, implying that the system simultaneously speculates all potential sequences—even though in reality, only one sequence occurs at a time. The system refines its guesses through subsequent observations, gradually eliminating incorrect anticipations until only one remains, thereby only one neuron in a *mini-column* being activated. This refinement process primarily relies on testing anticipations. When a neuron is activated, its connected neurons enter a *depolarized* state, if the relevant connections’ strengths are sufficient. When a *mini-column* is activated, neurons in the *depolarized* state are preferentially activated, while neurons in the *resting* state are inhibited. This *lateral inhibition* phenomenon is also observed in neuroscience^37^. Since only one event occurs at a time, most anticipations are inherently incorrect, with only the correct anticipation being retained. Consequently, most *depolarized* neurons revert to the *resting* state, while a few (often only one) *depolarized* neurons become *activated*. This process reduces multiple superposed sequences into a single correct one, functioning as a “filter” that gradually isolates the correct sequence.

Formally, let the *i*-th neuron in *mini-column* *c* be denoted by $N_c^{(i)}$, where the *mini-column* contains $n_c$ *neurons*. The activation rule for *mini-column* *c* is defined by Eq. (4),

4 $$\begin{aligned} A_c^{(i)} = \left\{ \begin{aligned} 1&~\text {if}&\forall j\in \{1,...,n_c\}, {\hat{A}}_c^{(j)}=0 \\ 1&~\text {if}&{\hat{A}}_c^{(i)}=1 \\ 0&~\text {if}&{\hat{A}}_c^{(i)}=0, \text {and}~ \exists j\in \{1,...,n_c\}, {\hat{A}}_c^{(j)}=1. \\ \end{aligned} \right. \end{aligned}$$

Here, $A_c^{(i)}$ indicates the *active*/*resting* state of neuron $N_c^{(i)}$, where $A_c^{(i)}=1$ indicates the neuron is in the *active* state and $A_c^{(i)}=0$ signifies the *resting* state. Meanwhile, ${\hat{A}}_c^{(i)}$ indicates the neuron’s *depolarized* state, with ${\hat{A}}_c^{(i)}=1$ indicating the *depolarized* state and ${\hat{A}}_c^{(i)}=0$ otherwise.

Neurons are interconnected through synapses, each exhibiting plasticity, meaning that its connection strength can change over time. Some learning rules regarding synaptic plasticity have been proposed, including *Hebbian* learning, Spike-Timing-Dependent Plasticity (STDP), etc^37^. If a synapse’s strength exceeds a predefined threshold (denoted by $\theta$), activation of the pre-synaptic neuron triggers activation of the post-synaptic neuron. Otherwise, the synapse is considered a *potential connection* waiting for being strengthened. The depolarization procedure is expressed by Eq. (5),

5 $$\begin{aligned} {\hat{A}}_{c_1}^{(i),t} = \left\{ \begin{aligned} 1&~\text {if}~ A_{c_2}^{(j),t-1}=1 ~\text {and}~ W_{c_1(i)}^{c_2(j)}>\theta \\ 0&~\text {otherwise}. \end{aligned} \right. \end{aligned}$$

Here, ${\hat{A}}_{c_1}^{(i),t}$ represents the *depolarized* state of neuron $N_{c_1}^{(i)}$ at timestep *t*. The condition $A_{c_2}^{(j),t-1}=1$ indicates that neuron $N_{c_2}^{(j)}$ was in the *active* state at timestep $t-1$, while $W_{c_1(i)}^{c_2(j)}$ denotes the synaptic strength connecting neuron $N_{c_1}^{(i)}$ to neuron $N_{c_2}^{(j)}$. Depolarization occurs if the pre-synaptic neuron was *active* in the previous timestep and the synaptic strength exceeds the threshold $\theta$; otherwise, the neuron remains in a non-depolarized state.

The specific rule governing synaptic strength modification is not detailed here. Generally, it follows principles similar to the *Hebbian* rule: a synapse is strengthened when both the pre-synaptic and post-synaptic neurons are activated simultaneously, and weakened when only one neuron is active within a brief time window. The learning mechanism employed in this study is a variant of the *Hebbian* rule, adapted to the proposed sequence learning framework.

Unlike the HTM theory^7^, where an event is represented by *sparse distributed representation*^7^, the model proposed in this study employs *concept-centered* representation. This approach enables the system to operate in a *human-understandable* manner, ensuring interpretability.

#### Logical representation

As previously mentioned, this paper seeks to establish a correspondence between *neurons* and *concepts*. Specifically, the *neuronal network* and the *conceptual network* can be described using distinct knowledge representations, yet they are fundamentally equivalent. The *conceptual network* seemed similar to traditional “semantic networks” and “knowledge graphs” but is essentially different. In traditional knowledge graphs, each node represents a concept, and edges represent relationships between concepts, akin to this model. However, traditional knowledge graphs are confronted with the symbol grounding problem and are often arbitrarily organized. Non-Axiomatic Logic (NAL) provides the theoretical foundation for the *conceptual network* described herein, offering a normative framework that governs the network’s self-organization process.

More specifically, a *mini-column* corresponds to a *concept*, which can have different meanings in different contexts. To capture this nuance, the model employs *contextual concepts*. When a *concept* is activated, all *contextual concepts* associated with it are evaluated to determine which specific meaning is contextually relevant. In the absence of contextual information, all corresponding *contextual concepts* are activated, implying that the system considers all possible sequences simultaneously while attempting to predict which *contextual concepts* will be activated next. Similar to the neural representation, when certain *contextual concepts* within a *concept* are anticipated, they are preferentially activated, whereas unanticipated *contextual concepts* are inhibited.

The prediction and activation of *contextual concepts* are governed by NAL. A connection between *contextual concepts* corresponds to a *predictive equivalence* *statement* in NAL. For example, a statement “*A* is of predictive equivalence to *B*” signifies a predictive relationship between *A* and *B*. The strength of the connection is quantified by the *truth-value* associated with the *statement*, which is updated as new evidence accumulates. Additionally, the activation state of *contextual concepts* is represented by corresponding *truth-values*. When a *contextual concept* *A* is activated, both *frequency* and *confidence* in its *truth-value* become high, with *confidence* decaying over time. In this model, *confidence* decays to zero after one timestep, implying the influence of an anticipation is transient. Considering a *predictive equivalence statement*, when *A* is activated, *B* is predicted with its *truth value* calculated based on the *deduction rule* of NAL. If *A* or *B* is activated, the *induction rule* of NAL is applied to accumulate evidence. The following provide a more formalized description.

The schematic diagram illustrating the representation approach is shown in Fig. 1a. A *column* is interpreted as a *concept*, within which several *nodes* exist. Each *node* represents a *contextual concept*, comprising a *truth-value* and relationships with other *contextual concepts*. The phrase “a (*contextual*) *concept* is activated” means that the system is perceiving or experiencing a specific stimulus at a given time. For example, when observing a red flower, the *concept* corresponding to the red flower is activated, meaning the system perceives the red flower. *Truth-value* consists of two parts, *frequency* (denoted as *f*) and *confidence* (denoted as *c*), expressed as a two-dimensional tuple $\langle f; c \rangle$. *Frequency* quantifies the proportion of positive evidence among all evidence, and *confidence* reflects the impact of future evidence. Notably, in NAL, there is no “absolute truth”; instead, the truth of a judgment is evaluated based on the evidence accumulated by the system. Let $w^+$ represent the amount of positive evidence and $w^-$ the amount fo negative evidence, making the total evidence *w*, defined as

$$\begin{aligned} w=w^++w^-. \end{aligned}$$

*Frequency* is calculated as

$$\begin{aligned} f=w^+/w, \end{aligned}$$

whereas *confidence* is determined by

$$\begin{aligned} c=w/(w+k), \end{aligned}$$

where *k* is a constant parameter. In this context, an *event* refers not to an external occurrence but to the subjective experience of that occurrence within the system.

In NAL, the temporal relations between two *concepts* $E_1$ and $E_2$ include *predictive implication* “$E_1 \mathrel {\Rightarrow \!\!\!\!\!\!\!/ \ }E_2$”, *retrospective implication* “$E_2 \mathrel {\Rightarrow \!\!\!\!\!\!\!\backslash \ }E_1$”, and *predictive equivalence* “$E_1 \mathrel {\Leftrightarrow \!\!\!\!\!\!/ \ }E_2$”. A sequence of *events* can be represented as “$(E_1, E_2,..., E_n)$”.

As depicted in Fig. 1a, a chain of nodes represents multiple beliefs simultaneously. For instance , “$A^{(1)} \mathrel {\Leftrightarrow \!\!\!\!\!\!/ \ }B^{(1)}$”, “$(A^{(1)}, B^{(1)}) \mathrel {\Leftrightarrow \!\!\!\!\!\!/ \ }C^{(3)}$”, and “$(A^{(1)}, B^{(1)}, C^{(3)}) \mathrel {\Leftrightarrow \!\!\!\!\!\!/ \ }D^{(4)}$” shares the same chain.

Consider two *events* “$E_1~\langle f_1; c_1 \rangle$” and “$E_2~\langle f_2; c_2\rangle$”, occurring at times $t_1$ and $t_2$, with $t_2 < t_1$, the *temporal induction rules* in NAL includes

6 $$\begin{aligned} \{ E_1~\langle f_1; c_1 \rangle , E_2.~\langle f_2; c_2 \rangle \}&\vdash E_2 \mathrel {\Rightarrow \!\!\!\!\!\!\!/ \ }E_1~\langle F_{ind} \rangle , \end{aligned}$$

7 $$\begin{aligned} \{ E_1~\langle f_1; c_1 \rangle , E_2.~\langle f_2; c_2 \rangle \}&\vdash E_1 \mathrel {\Rightarrow \!\!\!\!\!\!\!\backslash \ }E_2~\langle F_{ind}' \rangle , \end{aligned}$$

8 $$\begin{aligned} \{ E_1~\langle f_1; c_1 \rangle , E_2.~\langle f_2; c_2 \rangle \}&\vdash E_2 \mathrel {\Leftrightarrow \!\!\!\!\!\!/ \ }E_1~\langle F_{com} \rangle , \end{aligned}$$

where $F_{ind}$, $F_{ind}'$, and $F_{com}$ denote the *induction functions* that map the *truth-values* of the premises to those of the corresponding conclusions. The functions are defined as follows:

$$\begin{aligned} \langle F_{ind} \rangle \text {:}&~w^+=f_1f_2c_1c_2 ~\text {and}~ w=f_2c_1c_2\text {,}\\ \langle F_{ind}' \rangle \text {:}&~w^+=f_1f_2c_1c_2 ~\text {and}~ w=f_1c_1c_2\text {,}\\ \langle F_{com} \rangle \text {:}&~w^+=f_1 f_2 c_1 c_2 ~\text {and}~ w=(1-(1-f_1)(1-f_2))c_1c_2\text {.} \end{aligned}$$

*Frequency* and *confidence* are subsequently calculated from $w^+$ and *w*.

In this study, the *truth-value* of each *event* is assumed to be constant (e.g., $\langle 1.0;0.9 \rangle$), though they could be revised dynamically in future work. The *truth-value* of an anticipation can be derived by the *temporal deduction rule* in NAL, i.e.,

9 $$\begin{aligned} \{ E_1 \mathrel {\Leftrightarrow \!\!\!\!\!\!/ \ }E_2 ~\langle f_1; c_1 \rangle , E_1~\langle f_2; c_2 \rangle \} \vdash E_2~\langle F_{ded} \rangle , \end{aligned}$$

where $F_{ded}$ represents the *deduction function*, defined as

$$\begin{aligned} \langle F_{ded} \rangle \text {:} ~f=f_1 f_2 ~\text {and}~ c = f_1 f_2 c_1 c_2\text {.} \end{aligned}$$

When a *concept* is activated, that is, a corresponding *event* “$E \langle f;c \rangle$” occurs, relevant *contextual concepts* “$E^{(i)} \langle f^{(i)};c^{(i)} \rangle$” ($i\in {1,...,n}$, where *n* is the number of *contextual concepts* within *concept* *E*) are processed. If a *contextual concept* was anticipated previously and activated currently, the *revision* rule is applied to merge the two *truth-values*—one derived from the anticipated event and the other from the observed fact. The *revision* rule in NAL is

10 $$\begin{aligned} E \langle f_1;c_1 \rangle , E \langle f_2;c_2 \rangle \vdash E \langle F_{rev} \rangle , \end{aligned}$$

where $F_{rev}$ is *revision function*, defined as

$$\begin{aligned} \langle F_{rev}\rangle \text {:} ~ w^+=w_1^+ + w_2^+ ~\text {and}~ w^-=w_1^- + w_2^-. \end{aligned}$$

This indicates that an anticipated *event* attains higher *confidence* when when it is subsequently observed.

When a *concept* is activated, the selection of which *contextual concept* becomes active depends on the *expectations* associated with their *truth-values*. In NAL, the *expectation* of *statement* “$S \langle f;c \rangle$” is

11 $$\begin{aligned} e(S) = F_{exp}(f,c) = c(f-0.5)+0.5. \end{aligned}$$

This formulation integrates both *frequency* (*f*) and *confidence* (*c*) to quantify the likelihood of the statement’s truth, adjusted around a neutral baseline of 0.5.

A threshold $\zeta$ is introduced to determine activation conditions for *contextual concepts*. Specifically, if an *event* is observed but unanticipated, or if it is anticipated but unobserved, its *expectation* would be less than $\zeta$; conversely, if the *event* is anticipated and observed, its *expectation* would be greater than $\zeta$.

A *contextual concept* is activated if its *expectation* surpasses $\zeta$, *or* if all the *expectations* of *contextual concepts* in a *concept* are below $\zeta$. Formally,

12 $$\begin{aligned} A_c^{(i)} = \left\{ \begin{aligned} 1&~\text {if}&\forall j\in \{1,...,n_c\}, e(E_c^{(j)})<\zeta \\ 1&~\text {if}&e(E_c^{(j)})>\zeta \\ 0&~\text {if}&e(E_c^{(i)})<\zeta , \text {and}~ \exists j\in \{1,...,n_c\}, e(E_c^{(j)})>\zeta . \\ \end{aligned} \right. \end{aligned}$$

The procedure of *temporal induction* for *statement* “$E_{c_1}^{(i)} \mathrel {\Leftrightarrow \!\!\!\!\!\!/ \ }E_{c_1}^{(j)}$” happens only when $A_{c_1}^{(i)}=1$ or $A_{c_2}^{(j)}=1$, indicating that at least one of the corresponding *contextual concepts* is activated.

While both predictive implication (“$\mathrel {\Rightarrow \!\!\!\!\!\!\!/ \ }$”) and retrospective implication (“$\mathrel {\Rightarrow \!\!\!\!\!\!\!\backslash \ }$”) are important for comprehensive modeling, this study focuses primarily on *predictive equivalence* (“$\mathrel {\Leftrightarrow \!\!\!\!\!\!/ \ }$”) as a foundational approach for learning and inference.

#### Graph representation

As discussed above, the proposed model can be not only explained as a neuronal network but also interpreted by a logic. To further elucidate the model’s structure and avoid conceptual ambiguity, a more abstract representation is introduced, referred to as the graph representation, which leverages the formal language of *Graph Theory*. As illustrated in Fig. 1, the components of the graph are *nodes* and *links* (also known as vertices and edges in *Graph Theory*). A *column* (interpreted as hyper-vertex) is a collection of *nodes*. Each *link* possesses an adjustable weight, reflecting the strength of the connection between *nodes*. Each *node* can exist in one of the three states, *activation*, *non-activation*, and *pre-activation*. Each state is represented by a pair of real numbers (i.e., *truth-value* as defined previously), each ranging from 0 to 1.

The correspondence among terms across the three representations is summarized in Table 1.

**Table 1:** The correspondence of terms among the three representations: *Neural Representation*, *Logical Representation*, and *Graph Representation*.

Graph repr.	Neural repr.	Logical repr.
Node	Neuron	Contextual-concept
Column	Mini-column	Concept
Link	Synapse	Temporal statement (e.g., “$\langle A\mathrel {\Leftrightarrow \!\!\!\!\!\!/ \ }B\rangle$”)
Link-weight	Synaptic strength	Truth-value (*abbr.*, t.-v.)
Weight adjustment	Synaptic plasticity	Temporal-induction and revision
Activation	Active state	Event with t.-v. “(1.0; 0.9)”
Pre-activation	Depolarized state	Anticipation with t.-v. “(1.0; 0.9)”
Non-activation	Resting state	Event with t.-v. “(1.0; 0.1)”

### Sequence learning

A central challenge in this study is determining how to construct *links* given a sequence of *events*. Fully connecting all *nodes* (i.e., establishing links *between* every *node* pair) is infeasible, as it would lead to a combinatorial explosion of links. With an unbounded stream of events, the number of columns cannot be predefined, requiring the ability to dynamically generate new *columns*. Furthermore, a fully connected network would cause the number of *links* to increase exponentially with the number of columns. Due to the Assumption of Insufficient Knowledge and Resources (AIKR)^19^, the number of *links* connected to or from a *node* should not exceed a constant. This constraint necessitates a mechanism for creating new *links* while recycling old ones. Previouly, the logic rules of temporal *induction* has been introduced, however, when to perform *induction* and to revise the *link* remains to be addressed in the following.

#### Hypothesizing

Initially, the network contains neither *nodes* nor *links* When an *event* occurs, a corresponding *column* is constructed if it does not already exist. Each newly created *node* within a *column* initially has no *links*. When two *columns* are activated in succession, two corresponding sets of *nodes*—denoted as ${\mathscr {N}}_1$ in *column* ${C}_1$ and ${\mathscr {N}}_2$ in *column* ${C}_2$—are activated. From these sets, one *node* $E_{c_1}^{(i)}$ is selected from ${\mathscr {N}}_1$ and another *node* $E_{c_2}^{(j)}$ from ${\mathscr {N}}_2$. If a *link* connecting $E_{c_1}^{(i)}$ to $E_{c_2}^{(j)}$ does not already exist, a new one is established. The initial weight of this *link*, represented by its *truth-value*, is deliberately set to be low (according to the *induction* rule) to reflect uncertainty at the onset of learning. Such a *link*, expressed as“$E_{c_1}^{(i)} \mathrel {\Leftrightarrow \!\!\!\!\!\!/ \ }E_{c_2}^{(j)} \langle 1.0; 0.1 \rangle$”, embodies a *hypothesis*.

When selecting a *node* for *hypothesizing* from a set, the question arises: which node should be chosen? To answer this, we consider the *meaning* of a *node*. Since a *node* is activated within the context of specific *events*, it can be understood as representing a concept under certain contextual conditions. Ideally, a node should have at most one *pre-link* (i.e., an incoming link), at most one *post-link* (i.e., an outgoing link), and at least one pre- or post-link to insure proper connectivity with other concepts. Under these conditions, a *node* becomes activated if and only if a specific event context occurs. For instance, consider a *node* $B^{(1)}$ that activates exclusively in the sequence “(*A*, *B*, *C*, *D*)”. Here, $B^{(1)}$ uniquely identifies the *event* *B* in that sequence, rather than the *event* *B* in “(*X*, *B*, *C*, *Y*)”. However, the AIKR mandates that the number of *nodes* remain constant. Consequently, each *node* must be capable of representing multiple contexts. The meaning of a *node* is considered clear or unambiguous *either if* (1) one *post-link*’s strength significantly exceeds that of other *post-links*, and (2) one pre-link’s strength significantly surpasses that of other *pre-links*, *or if* (3) either a single *pre-link* or *post-link* dominates all other links. To minimize disruption of the *nodes* with clear meanings during *hypothesizing*, the guiding principle is to select the *node* with the lowest *utility*. Here, the *utility* of node $E_c^{(i)}$ is defined as

13 $$\begin{aligned} u(E_c^{(i)}) = 1-(1-u_1)(1-u_2), \end{aligned}$$

where

14 $$\begin{aligned} \begin{aligned} u_1&= \left\{ \begin{aligned}&0, & ~\text {if}~{\mathscr {L}}_{pre}(E_c^{(i)}) = \varnothing \\&\max _{\forall L \in {\mathscr {L}}_{pre}(E_c^{(i)})} e(L), & ~\text {otherwise} \end{aligned} \right. , ~\text {and} \\ u_2&= \left\{ \begin{aligned}&0, & ~\text {if}~{\mathscr {L}}_{post}(E_c^{(i)}) = \varnothing \\&\max _{\forall L \in {\mathscr {L}}_{post}(E_c^{(i)})} e(L), & ~\text {otherwise} \end{aligned} \right. \end{aligned} \end{aligned}$$

Here, ${\mathscr {L}}_{pre}(E_c^{(i)})$ and ${\mathscr {L}}_{post}(E_c^{(i)})$ denote the sets of *pre-links* and *post-links* associated with *node* $E_c^{(i)}$, respectively. The function *e*(*L*) represents the *expectation* of the *truth-value* of *link* *L* (refer to Eq. (11)). If a *node* possesses a clear and unambiguous meaning, it is generally less likely to be selected for *hypothesizing*.

New *hypotheses* are continuously generated; however, they do not yield strong conclusions until sufficient evidence is accumulated. For instance, a weak *hypothesis*, such as “$E_{c_1}^{(i)} \mathrel {\Leftrightarrow \!\!\!\!\!\!/ \ }E_{c_2}^{(j)} \langle 1.0; 0.1 \rangle$”, fails to activate its consequent *node* $E_{c_2}^{(j)}$, as described in Eq. (12). In this sense, a hypothesis represents a *potential predictive relation* between two nodes. Such a relation becomes effective for forecasting only when its strength exceeds a predefined threshold. Notably, a *link* can be reinforced regardless of its initial strength. This means that even a weak *hypothesis* can gradually strengthen through repeated activation of its antecedent and consequent.

#### Revising

Whenever a *column* is activated, a *link* is selected for revising, if a known cause can be determined. The guiding principle is to enhance the *link* that is most likely to become conclusive. The guiding principle is to enhance the *link* that is most likely to become conclusive. Formally, the selected *link* *L* is

15 $$\begin{aligned} L=\underset{{\forall L\in {\mathscr {L}}_{pre}(E_{c}^{(i)}),\forall i \in \{1,...,n_c\}}}{\textrm{argmax}}e(L), \end{aligned}$$

which indicates that the algorithm selects the *link* with the maximum *expectation* among all the *pre-links* associated with *nodes* in the activated *column*. As a result, a *link* represented by statement “$E_{c_1}^{(i)} \mathrel {\Leftrightarrow \!\!\!\!\!\!/ \ }E_{c_2}^{(j)}$” is selected for *revising*.

Given two nodes, $E_{c_1}^{(i)}$ and $E_{c_2}^{(j)}$, the temporal *induction* rule is employed to update the *truth-value* of the statement “$E_{c_1}^{(i)} \mathrel {\Leftrightarrow \!\!\!\!\!\!/ \ }E_{c_2}^{(j)}$” (refer to Eqs. (8) and (10)). A distinguishing feature of this learning procedure is that negative evidence is generated when an anticipated event fails to occur.

Specifically, the consequent *node* $E_{c_2}^{(j)}$ enters the *pre-activation* state at timestep *t* (i.e., $\hat{A}_{c_2}^{(i), t}=1$) if both of the following conditions are satisfied: (1) The antecedent *node* $E_{c_1}^{(i)}$ is in the *activation* state at timestep $t-1$ (i.e., $A_{c_1}^{(i),t-1}=1$), and (2) the *expectation* of the corresponding statement exceeds a predefined threshold $\theta$ (*i.e.*, $e(E_{c_1}^{(i)} \mathrel {\Leftrightarrow \!\!\!\!\!\!/ \ }E_{c_2}^{(j)}) > \theta$). Formally,

16 $$\begin{aligned} \hat{A}_{c_2}^{(i), t} = \left\{ \begin{aligned}&1, & ~\text {if}~ A_{c_1}^{(i),t-1}=1, ~\text {and}~ e(E_{c_1}^{(i)} \mathrel {\Leftrightarrow \!\!\!\!\!\!/ \ }E_{c_2}^{(j)}) > \theta&\\&0, & ~\text {otherwise}&\end{aligned} \right. \end{aligned}$$

While an *event* may generally have multiple causes and effects, the proposed sequence learning model simplifies this complexity by constructing *causal chains*, where each *event* has at most one cause and one effect within a given context.

Consider the scenario where $\exists j\in \{1,...,n_c\}~\text {such that}~e(E_c^{(j)})>\zeta$, which implies that the current *event* has a specific context where some—but not all—*nodes* within a *column* are activated. If a *node* $E_{c_2}^{(j)}$ is anticipated (i.e., $\hat{A}_{c_2}^{(i), t}=1$) but not activated (i.e., ${A}_{c_2}^{(i), t}=0$), negative evidence is collected for $E_{c_2}^{(j)}$. Conversely, when a *node* $E_{c_2}^{(j)}$ is activated, the model examines all its possible antecedents through the *pre-links*. If an antecedent *node* $E_{c_1}^{(i)}$ was not activated prior to $E_{c_2}^{(j)}$, despite the presence of a corresponding statement “$E_{c_1}^{(i)} \mathrel {\Leftrightarrow \!\!\!\!\!\!/ \ }E_{c_2}^{(j)}$”, negative evidence is collected for $E_{c_2}^{(j)}$. When the two *nodes*, $E_{c_1}^{(i)}$ and $E_{c_2}^{(j)}$, are activated in succession, the model directly applies the temporal *induction* and the *revision* rules to update the corresponding *truth-value*.

In practical implementation, a simplified yet equivalent approach is adopted: if both nodes are activated in succession, a positive evidence amount is collected ($w^+=p^+$); If only one node is activated while the other is not, negative evidence is gathered ($w^-=p^-$). Here, $p^+$ and $p^-$ are hyper-parameters of the model, typically set to $p^+=p^-=1$, controlling the degree of evidence accumulation. A penalty mechanism is also introduced for scenarios where $\forall j\in \{1,...,n_c\}~\text {such that}~e(E_c^{(j)})<\zeta$, indicating that all *nodes* within a *column* are *inactive* without prior anticipation. In such cases, the entire *column* becomes activated, triggering a multiple anticipations. For those anticipations that are not subsequently verified, a small amount of negative evidence is accumulated, calculated as $w^-=|\{e(L)>\theta ,\forall L\in {\mathscr {L}}_{post}(E_{c_1}^{(i)})\}|/b$: the “$|\{\cdot \}|$” term denotes the number of *post-links* from *node* $E_{c_1}^{(i)}$, with link *expectation* exceeding the threshold $\theta$, and *b* is a constant (e.g., $b=40$). Both $\theta$ and *b* are hyper-parameters of the model. Notably, when $b=\infty$, no penalty is imposed, allowing the model to disregard unverified anticipations.

#### Recycling

Due to AIKR, the number of *links* associated with a given *node* must not exceed a predefined threshold. When this limit is surpassed, excess *links* are removed. This mechanism mirrors the forgetting process observed in biological memory systems, enabling the model to prioritize strong connections while discarding less significant ones.

The *pre-links* ${\mathscr {L}}_{pre}(E_{c_1}^{(i)})$ and *post-links* ${\mathscr {L}}_{post}(E_{c_1}^{(i)})$ associated with *node* $E_{c_1}^{(i)}$ are organized within a priority queue, where links are ranked based on their *utility*. In the current design, the *utility* of a *link* is directly determined by the *expectation* of its *truth-value* (refer to Eq. 11):

17 $$\begin{aligned} u(L) = e(L). \end{aligned}$$

This design ensures that links with higher *expectations*—and thus greater predictive relevance—are given higher priority within the network’s structure.

When the number of *link*s, denoted as $n_L$, in the priority queue exceeds a predefined threshold, denoted as $\xi$, the model recycles the exceeding links. Specifically, the $(n_L-\xi )$ *links* with the lowest priorities (i.e., those with the smallest utility values) are deleted. This process allows the model to dynamically adapt to new information while maintaining a bounded network capacity, and preventing over-processing of outdated or infrequent patterns.

## Data Availability

The datasets generated and analyzed during the current study are available in the “SeL-NAL” repository, https://github.com/bowen-xu/SeL-NAL.
